# Supplementary material for: Effects of Impregnated Amidophosphonate Ligand Concentration on the Uranium Extraction Behavior of Mesoporous Silica
Source: Molecules. 2022 Jul 6;27(14):4342. doi: 10.3390/molecules27144342 (PMC9316337; doi:10.3390/molecules27144342)
Supplement: Supplementary file 1 [file molecules-27-04342-s001.zip › molecules-1755989-supplementary.pdf]

Supplementary Materials

Effects of Impregnated Amidophosphonate Ligand Concentration on the Uranium Extraction Behavior of Mesoporous Silica

Aline Dressler, Antoine Leydier * and Agnès Grandjean

CEA, DES, ISEC, DMRC, Univ Montpellier,34000 Marcoule, France; aline.dressler@cea.fr (A.D.); agnes.grandjean@cea.fr (A.G.)
* Correspondence: antoine.leydier@cea.fr; Tel.: +33-46-633-9474

I Materials

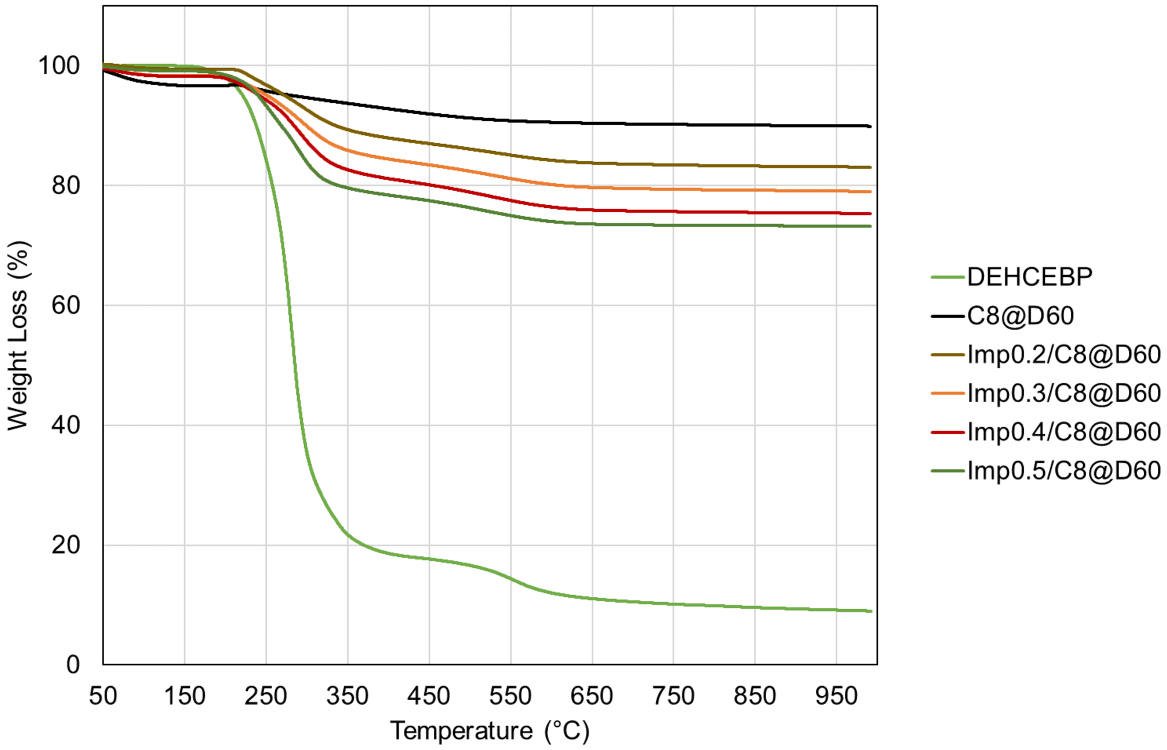

Figure S1. Thermal stability of the amidophosphonate ligand (DEHCEBP), the prefunctionalized support (C_8@D60) and the impregnated materials with different DEHCEBP loadings (0.2–0.5 $mmol \cdot g^{-1}$) between 50 and 1000 °C.
